# Validity and reliability of a physical activity questionnaire for Vietnamese adolescents

**DOI:** 10.1186/1479-5868-9-93

**Published:** 2012-08-01

**Authors:** Tang K Hong, Nguyen HHD Trang, Hidde P van der Ploeg, Louise L Hardy, Michael J Dibley

**Affiliations:** 1 Department of Community Health, Pham Ngoc Thach University of Medicine, Ho Chi Minh City, Vietnam; 2 Sydney School of Public Health, Sydney Medical School, University of Sydney, NSW, 2006, Australia; 3 Cluster for Physical Activity and Health, Sydney School of Public Health, University of Sydney, NSW, 2006, Australia; 4 Prevention Research Collaboration, Sydney School of Public Health, University of Sydney, NSW, 2006, Australia

**Keywords:** Reliability, Validity, Physical activity questionnaire, Adolescents, Vietnam

## Abstract

**Background:**

Accurate assessment of physical activity in adolescents at population level is necessary. In Vietnam, the International Physical Activity Questionnaire (IPAQ) and Physical Activity Questionnaire for Adolescents (PAQA) have been validated against accelerometers for use in adolescents. However, these questionnaires were originally designed for adults and showed poor validity. This study aims to assess the reliability and validity of the Vietnamese Adolescent Physical Activity Recall Questionnaire (V-APARQ).

**Methods:**

One hundred and sixty five students were recruited from four junior high schools in Ho Chi Minh City Vietnam in 2004. V-APARQ asked students to report their usual organised and non-organised physical activity during a normal week and moderate- (MPA), vigorous- (VPA and moderate-to-vigorous- (MVPA) physical activity were calculated. Reliability was assessed by test-retest (2 weeks apart). Construct validity was assess by 7-day accelerometry, following the completion of the first V-APARQ.

**Results:**

The construct validity of the V-APARQ showed Spearman correlation of 0.25 and 0.22 for the assessment of the questionnaire when compared to the accelerometer. Test-retest reliability showed a weighted Kappa of 0.75 and the intra-class correlation coefficient for MVPA was 0.57 for the whole group (MPA =0.37 and VPA = 0.62), and were higher in boys than girls. The Bland-Altman plots for reliability show a mean difference of 0.4 minutes (95 % CI = −3.2, 4.0) for daily MVPA (n = 146) and the limits of agreement were −42.6 to 43.4 mins/day. In boys MVPA was lower on the first, compared with second administration of V-APARQ while the reverse was observed among girls.

**Conclusion:**

The reliability and validity of the V-APARQ were low to fair, but are comparable to other self-report physical activity questionnaires used among adolescents. V-APARQ will be useful for population monitoring of change in physical activity among urban Vietnamese adolescents.

## Background

Physical activity across the life span is an important component of a healthy lifestyle, and the benefits of being active to prevent a range of chronic diseases and obesity are well documented [1-3]. For youth aged 5–18 years, it is recommended that they should participate daily in 60 minutes or more of moderate to vigorous physical activity (MVPA) for health [2,3]. However, the evidence suggests few adolescents meet the guideline [4,5]. Thus, assessment of physical activity in adolescents at population level is needed to identify current levels of physical activity, provide a basis for targeted intervention, and assess the effectiveness of intervention programs designed to increase physical activity.

A wide range of objective and subjective methods have been used to measure physical activity behavior among children and adolescents. Due to their low cost and ease of administration, self-report methods are the most commonly used in population monitoring of physical activity, but are prone to recall and social desirability bias [6]. Hence, questionnaires should be validated against more objective measures of physical activity before use. In Vietnam, the International Physical Activity Questionnaire (IPAQ) and Physical Activity Questionnaire for Adolescents (PAQA) have been validated against accelerometers for use in Vietnamese adolescents. However, these questionnaires were originally designed for adults and showed “poor validity to be used as a population instrument in Vietnam” [7]. The Adolescent Physical Activity Recall Questionnaire (APARQ), which has been developed and validated among adolescents in Australia [8,9], was considered a potentially acceptable self-report questionnaire for Vietnamese youth. The purpose of this study was to determine the test–retest reliability and construct validity of a translated and culturally adapted version of APARQ (i.e., Vietnamese-APARQ or V-APARQ) for assessing MVPA among Vietnamese youth.

## Methods

### Study design

A convenience sample of adolescents who were participating in a longitudinal study of school students (Ho Chi Minh City Youth Cohort Study, HCMC-YCS) were recruited. Reliability of the V-APARQ was assessed using two week test-retest and the construct validity was determined by accelerometry, which was worn between the first and second administration of the V-APARQ.

### Participant recruitment

Four junior high schools (Grades 6 – 9) were selected at random from schools participating in the HCMC-YCS in 2004 [10]. Two schools were based in wealthy urban districts and two from less wealthy urban districts. In each selected school, one class of Grade 6 was chosen and students were invited to participate in the study. In total, 165 students from the four schools were sampled. The study was approved by the Ethics Committee of Pham Ngoc Thach University of Medicine. Written consent was obtained from both students and their parents prior to data collection.

### Outcome assessment

The APARQ [8] was adapted and translated into Vietnamese. Briefly, APARQ asks students to think about a normal week during summer school and winter school terms and report separately time spent in organized and non-organized physical activities. Students report the activity, the duration and frequency for each activity reported. In order to ensure APARQ was suitable for Vietnamese youth, a modified version of APARQ was guided by formative research. This formative research comprised focus group discussions among adolescents, and physical education teachers from 30 junior high schools of different areas in Ho Chi Minh City to identify the range of physical activities that needed to be considered in the questionnaire. The purpose of these discussions was to make sure the list of common activities was appropriate for use with Vietnamese adolescents. After group discussions, a list of common activities done by junior high school students was created and added to the APARQ as prompts to help the students recall their activities. The revised questionnaire was pilot tested in approximately 20 junior high schools (n = 96) prior to the validation study. Some minor changes were made based on the pilot. For example, the form included some open-ended questions for physical activities not included in the list. Furthermore, the questionnaire also included mode and time in commuting to school.

In Ho Chi Minh City, because of the tropical environment, there is no clear difference between the summer and winter school terms. Hence, the original APARQ questionnaire was modified and asks students to report time spent in organised and non-organised sports and games at school, before and after school and on weekends during both the school term and the summer holidays. The questions on frequency and duration of each activity were administered during class time with the researcher providing prompts. The interviewers helped the students to remember activities they have engaged in at school, at home, during school time and summer holidays by following a suggested list of common activities at the end of the V-APARQ. The interviewers also helped the students to assign activities into organized and non-organized columns, and to confirm if the activities were in training or in competition. Four interviewers administered the V-APARQ during class time and assisted the students to answer the questionnaires.

Physical activity was also assessed by an Actigraph GT1M accelerometer (Actigraph, LLC, Pensacola, FL, USA), an accepted construct measure of physical activity [11]. The Actigraph accelerometers were initialised to collect data in 1-minute epochs and students were asked to wear the accelerometer on the right hip for seven consecutive days, except during sleep and water based activities.

### Data management and analysis

Data on self-reported physical activity were cleaned based on the guidelines of the original APARQ [8]. Calculations were conducted separately for school term and summer holidays and for organised and non-organised activities. Each activity was categorized as a moderate physical activity (MPA) or vigorous physical activity (VPA) based on the Compendium of Physical Activities [12,13]. In Vietnam the school term is 38 weeks and summer holidays 14 weeks, so responses were weighted as; time spent in each activity per week = (Time spent in each activity per week in school term * 38/52) + (Time spent in each activity per week in summer holidays * 14/52).

For the accelerometer, average time spent per day in MPA (≥3 MET and <6 MET), VPA (≥6 MET) and moderate-to-vigorous physical activity (MVPA) (≥3 MET) were calculated using existing age specific cut points for accelerometer activity counts [14,15]. Non-wear time was defined as 10 minutes of consecutive zeroes [16]. Only participants who wore the accelerometer for eight or more hours per day on at least four out of seven days were included in the analysis.

MVPA was calculated in minutes per day for both the V-APARQ and accelerometer. As the distributions of the physical activity data were skewed, the median and interquartile range were presented. For validity, the differences between the two methods were tested using Mann–Whitney test. Spearman’s coefficients were calculated to assess the construct validity correlation between time (minutes per day) spent in MPA, VPA, and MVPA from each of the two V-APARQ assessments and the accelerometer. We also assessed the agreement of quintiles of time spent in MPA, VPA and MVPA based on the two measures using quadratic weighted Kappa statistics. The weighted Kappa was interpreted as follows: < 0.20: Slight agreement, 0.21– 0.40: Fair agreement, 0.41–0.60: Moderate agreement, 0.61–0.80: Substantial agreement, 0.81–0.99: Almost perfect agreement [17]. Bland-Altman plots were used to evaluate the level of agreement between the two V-APARQ measurements [18,19]. The data were analysed using STATA version 12 (Stata Corporation statistical packages, 2011).

## Results

A total of 165 students participated in the study, and 146 students (88%) had complete data (47% male). Mean and standard deviation of students’ age, weight, height and body mass index of the participants were 12.7 years (± 0.6), 40.4 (± 9.1) kg, 147.4 (± 7.7) cm and 18.9 (± 3.0) kg/m^2^, respectively.

### Validity

Table 1 shows descriptive data from the accelerometer and the V-APARQ, stratified by sex. Overall, the median of time reported for MPA was approximately 70 ~ 80 minutes and 11 minutes for VPA on the V-APARQ, which was significantly more than the median time measured by the accelerometer (57 minutes and 3 minutes, respectively) (p < 0.001). Boys spent more time in MPA and VPA, measured by V-APARQ and accelerometer, compared with girls*.*

**Table 1:** Median and inter-quartile range [IQR] of students’ MPA, VPA, and MVPA (mins/day) by instrument and sex (n = 146)

	**All (n = 146)**			**Boys (n = 69)**			**Girls (n = 77)**		
	**Accelerometers**	**V-APARQ1**^**a**^	**V-APARQ2**^**b**^	**Accelerometers**	**V-APARQ1**^**a**^	**V-APARQ2**^**b**^	**Accelerometer**	**V-APARQ1**^**a**^	**V-APARQ2**^**b**^
	**Median**	**Median**	**Median**	**Median**	**Median**	**Median**	**Median**	**Median**	**Median**
	**[IQR]**	**[IQR]**	**[IQR]**	**[IQR]**	**[IQR]**	**[IQR]**	**[IQR]**	**[IQR]**	**[IQR]**
*MPA (min/d)*	57.4	80.4	72.4	74.7	127.7	130.1	42.5	48.2	50.2
	[40.4, 75.0]	[50.4, 124.8]	[40.9, 174.1]	[63.0, 92.6]	[106.8, 160.6]	[39.9, 178.1]	[29.3, 57.0]	[19.8, 64.8]	[29.8, 57.5]
*VPA (min/d)*	3.0	10.4	10.1	7.8	11.3	14.6	1.6	10.4	7.8
	[1.4, 7,8]	[8.2, 14.3]	[6.6, 13.9]	[3.8, 14.8]	[3.2, 21.9]	[14.3, 52.9]	[0.8, 2.8]	[6.7, 15.9]	[5.0, 15.0]
*MVPA (min/d)*	61.3	90.8	82.3	83.4	139.5	144.6	45.0	58.8	58.1
	[41.8, 84.0]	[56.0, 137.8]	[56.3, 139.4]	[69.0, 109.3]	[126.5, 157.2]	[125.4, 167.4]	[29.8, 57.5]	[40.6, 74.4]	[47.2, 73.2]

^**a**^** = first administration of V-APARQ.**^**; b**^** = second administration of V-APARQ.**

Table 2 shows the Spearman correlation coefficients of time spent in MPA, VPA and MVPA measured by the accelerometers and for each administration of V-APARQ by sex. There were no differences in the correlation coefficients between accelerometer and the first and second administrations of V-APARQ. Overall, the correlation coefficients for MVPA were 0.25 and 0.22 for the first and second administrations, respectively, compared with accelerometry. Correlation coefficients were stronger for VPA compared with MPA and MVPA, and the coefficients were higher among boys compared with girls.

**Table 2:** Correlation coefficients and differences of time spent in MPA, VPA and MVPA measured by accelerometer and the V-APARQ

	**Accelerometer vs V-APARQ1**			**Accelerometer vs V-APARQ2**			**Differences between accelerometers and V-APARQs**		
	**All**	**Boys**	**Girls**	**All**	**Boys**	**Girls**	**All**	**Boys**	**Girls**
	**(n = 146)**	**(n = 69)**	**(n = 77)**	**(n = 146)**	**(n = 69)**	**(n = 77)**	**Mean**	**Mean**	**Mean**
							(95 % CI)	(95 % CI)	(95 % CI)
*MPA*	0.19*	0.24*	0.10*	0.18*	0.25*	0.12*	30.2	60.7	3.2
							(22.7, 37.6)	(50.1, 71.4)	(0.0, 8.6)
*VPA*	0.50*	0.52*	0.48*	0.48*	0.52*	0.43*	4.3	0.2	7.6
							(2.0, 6.6)	(0.0, 4.3)	(5.3, 9.8)
*MVPA*	0.25*	0.30*	0.18*	0.22*	0.33*	0.15*	35.9	61.3	10.1
							(23.3, 48.4)	(40.9, 87.6)	(5.8, 20.4)

* Spearman correlation coefficients significantly different from zero (p < 0.05).

** Differences between of accelerometers and the average of V-APARQs.

### Reliability

Two week test-re-test (V-APARQ1 and V-APARQ2) showed that the median time (mins/day) spent in MPA, VPA and MVPA were not significantly different (p-values = 0.44, 0.98, 0.88, respectively). Intraclass correlation coefficients ranged from 0.37 (MPA) to 0.62 (VPA) and 0.57 (MVPA), and were higher in boys than in girls. Weighted Kappa values showed moderate agreement for MVPA (Table 3).

**Table 3:** Test-retest reliability (intraclass correlation coefficients - ICC); percent agreement and weighted Kappa of quintiles of time spent in MPA, VPA and MVPA, measured by V-APARQ assessments in a sample of 146 junior high school students

	**ICC**			Percent agreement**			Weighted Kappa** (95 % CI)		
	**All**	**Boys**	**Girls**	**All**	**Boys**	**Girls**	**All**	**Boys**	**Girls**
	**(n = 146)**	**(n = 69)**	**(n = 77)**	**(n = 146)**	**(n = 69)**	**(n = 77)**	**(n = 146)**	**(n = 69)**	**(n = 77)**
*MPA*	0.37*	0.45*	0.30*	83.4	83.8	82.8	0.33	0.34	0.33
							(0.28, 0.35)	(0.25, 0.50)	(0.29, 0.40)
*VPA*	0.62*	0.65*	0.60*	88.0	87.8	88.2	0.52	0.58	0.45
							(0.42, 0.57)	(0.43, 0.72)	(0.36, 0.58)
*MVPA*	0.57*	0.70*	0.53*	93.8	91.8	92.4	0.75	0.78	0.70
							(0.74, 0.78)	(0.32, 0.85)	(0.30, 0.74)

* The % agreement and Kappa are based on quintiles.

**Figure 1:**
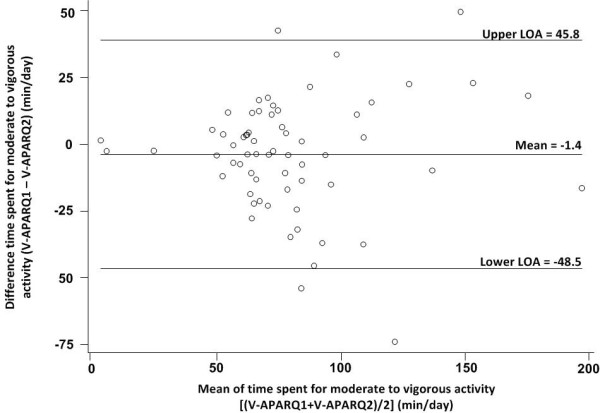
Bland-Altman plots for time spent in MVPA (mins/day) for boys (n = 69).

**Figure 2:**
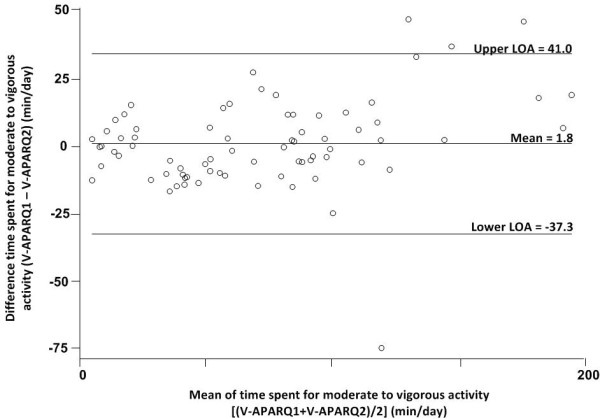
Bland-Altman plots for time spent in MVPA (mins/day) for girls (n = 77).

The Bland-Altman plots show that among boys, MVPA was lower on the first, compared with second administration of V-APARQ while the reverse was observed among girls. In boys, the higher the values reported in the V-APARQ1, the larger were the errors between two V-APARQs, but this pattern was not seen for girls. (Figure 1 and 2).

## Discussion

The reliability and validity of the V-APARQ were low to fair, but are comparable to other self-report physical activity questionnaires used among adolescents. V-APARQ will be useful for population monitoring of change in physical activity among Vietnamese youth.

### Validity

While the correlation coefficients reported here were small, they are consistent with the psychometric properties reported for the original APARQ [8] and by others for self-report questionnaires for physical activity among adolescents [20]. For example, the study in Canada using Caltrac motion sensor had the correlation coefficient at 0.33 [21] and the HELENA study conducted in Europe using Actigraph GT1M accelerometers had correlation coefficient at 0.25-0.30 for MPA and VPA of the group aged 15–17 [22]. Another study conducted on Vietnamese adolescents assessing the construct validity of two questionnaires IPAQ and PAQA also showed the correlation coefficients of 0.21 and 0.27 for urban areas.

In this study, the correlation coefficients found were higher in VPA than in MPA when comparing the data obtained with V-APARQ and those from the accelerometers. It has been reported in many studies that low and moderate intensity activities are the most difficult for adolescents to recall accurately because these activities are accumulated throughout the day and are diverse resulting in poorer recall [23,24]. In contrast, vigorous intensity physical activities are more structured and stable over time and easier to recall [25].

Our study found that, on average, children overestimated the time they spent in MPA by 60 min/day and the time they spent in VPA approximately by 7 min/day. These findings are consistent with other studies that have shown that VPA is typically reported with lower error [22,26].

### Reliability

The test-retest reliability correlation coefficients in the present study gave similar values as seen in other reliability studies [8,27], especially the original APARQ [8]. The overall ICC of 0.57 for MVPA indicates a negative score for test-retest reliability as suggested by Chinapaw et al. [28]. It is possible that the activities reported changed during the two-week test-retest period resulting in a lower reliability.

The greater reliability coefficients for VPA compared with MPA could indicate better recall of vigorous activities. Other studies conducted in adolescents also found that reliability for estimations of time spent on VPA was better compared to MPA [7,22]. However, this could also partially be explained by the notion that VPA are more fixed activities whereas the MPA are more variable [27]. It is important to note that the 2-week test-retest to assess the questionnaire’s reliability will likely have picked up some actual variations in activities between the two assessments periods, which would have led to an underestimation of the reliability coefficient. The Bland–Altman plots further suggested that the V-APARQ has low reliability for MVPA in both boys and girls. The limits of agreement (LOA) are indicating that some subjects under- or over-reported their time spent for MVPA in V-APARQ2 compared to V-APARQ1 with a difference observed between boys and girls. . It is difficult to determine if this reflects actual differences in physical activity behavior or is reflective of the measurement properties of the V-APARQ. It might be at least partly due to actual differences in physical activity behavior, as the second administration of the V-APARQ happened during a school exam period, while the first was during a regular school period. Hence, the balance between physical activities and study time (including after class) could have been quite different.

A limitation of the study is the lack of consensus on cut points for MVPA among youth, which makes it hard to determine absolute validity. However, accelerometers are arguably the most appropriate construct measure to determine the validity of assessing adolescent physical activity. Limitations of accelerometer are that they underestimate physical activities without a strong vertical component (such as cycling) and that they were not worn during aquatic activities [29,30]. In the current study, activity counts were collected in 1 minute epochs and this might have underestimated high level intensity activities of short duration. This is known to lead to underestimations of physical activity in young children (i.e., <5 years old), in which data collection in 15 second epochs is recommended [31]. However, this is less likely to be a major issue in adolescent populations, where physical activity bouts are maintained for longer periods compared with pre-school aged children.

Intra-interviewer variability was not measured in this study, however interviewers were trained in the administration of the V-APARQ so that the administration would be consistent. Another limitation was that the study only included adolescents in urban areas and it is yet to be determined if the V-APARQ will be as accurate when applied to rural areas in Vietnam. Thus, the tool should be re-validated before being used in a rural setting.

One of the limitations in categorising physical activities reported by youth is the lack of a compendium which defines MET values for a range of activities. Current practice has been to apply adult MET values [13]. This practice has been examined and efforts to build a physical activity compendium for youth were explored [32]. However, the proposed youth compendium consists mainly of adult values due to a lack of energy cost studies (only 35 % of the values listed in the compendium are based on data measured in youth, the rest are estimated from the adult compendium). Furthermore, it has been proposed that the cut-point to define MPA among youth should be raised from 3 to 4 METS [32], but to date redefining MPA is yet to be internationally agreed upon. To this end, we believe that our definition and MET allocations are appropriate for this validation study.

## Conclusions

In conclusion, the results of this study overall suggest that the V-APARQ has low to fair reliability and validity. It can be considered as useful measure of physical activity as well as monitor change and trends in physical activity among urban Vietnamese adolescents. Further validation in rural Vietnamese adolescents is advisable.

## Abbreviations

APARQ: Adolescent Physical Activity Recall Questionnaire; HCMC-YCS: Ho Chi Minh City Youth Cohort Study; ICC: Intraclass correlation coefficients; IPAQ: International Physical Activity Questionnaire; MPA: Moderate physical activity; MVPA: Moderate to vigorous physical activity; PAQA: Physical Activity Questionnaire for Adolescents; VPA: Vigorous physical activity; V-APARQ: Vietnamese-Adolescent Physical Activity Recall Questionnaire.

## Competing interests

No author has any financial and personal relationships with other people or organisations that could inappropriately influence (bias) our work.

## Authors’ contributions

TKH conducted data analysis and prepared the manuscript. NHHDT was responsible for management of data collection and entry and contributed part of data analysis. HvdP analysed data of accelerometers and also contributed to manuscript revision. LH provided advice about APARQ and the analysis of data and contributed to the manuscript revision. MD partly contributed to the analysis of data and preparation of the manuscript. No author has any financial or personal relationships with the organization sponsoring this research. The corresponding author has full access to all the data in the study and had final responsibility for the decision to submit for publication. All authors read and approved the final manuscript.
